# Neutralizing Antibody Response to BBIBP-CorV in Comparison with COVID-19 Recovered, Unvaccinated Individuals in a Sample of the Pakistani Population

**DOI:** 10.3390/vaccines10050692

**Published:** 2022-04-28

**Authors:** Javeria Aijaz, Shakir Hussain, Fouzia Naseer, Fatima Kanani, Sabiha Anis, Samreen Sarfaraz, Saima Saeed, Hina Farooq, Saba Jamal

**Affiliations:** 1 Molecular Biology Section, Pathology Department, Indus Hospital & Health Network, Karachi 75190, Pakistan; shakir.hussain@tih.org.pk (S.H.); fouzia.naseer@tih.org.pk (F.N.); 2 Chemical Pathology Section, Pathology Department, Indus Hospital & Health Network, Karachi 75190, Pakistan; fatima.kanani@tih.org.pk (F.K.); hina.farooq@tih.org.pk (H.F.); 3 Immunology Section, Pathology Department, Indus Hospital & Health Network, Karachi 75190, Pakistan; sabiha.anis@tih.org.pk; 4 Infectious Diseases Department, Indus Hospital & Health Network, Karachi 75190, Pakistan; samreen.sarfaraz@tih.org.pk; 5 Pulmonology Department, Indus Hospital & Health Network, Karachi 75190, Pakistan; saima.saeed@tih.org.pk; 6 Pathology Department, Indus Hospital & Health Network, Karachi 75190, Pakistan; saba.jamal@tih.org.pk

**Keywords:** BBIBP-CorV, Sinopharm, COVID-19, Pakistan, pseudotyped, neutralizing, immunoassay, vaccine

## Abstract

Fifty five percent of the Pakistani population is still unvaccinated with the two-dose protocol of COVID-19 vaccines. This study was undertaken to determine the seroconversion rate and antibody titers following the two-dose BBIBP-CorV protocol, and to compare these variables in unvaccinated, COVID-19 recovered individuals (total *n* = 180) at Indus Hospital and Health Network, Karachi. Pseudotyped lentivirus antibody neutralization assays and SARS-CoV-2 IgG Quant II (Abbott) immunoassays were performed 4-8 weeks following the second dose of the BBIBP-CorV or PCR positivity/onset of symptoms of COVID-19. Seroconversion rate, using neutralization assays, in vaccinated individuals was lower (78%) than that in unvaccinated, COVID-19-recovered individuals with moderate to severe infection (97%). Prior PCR positivity increased serocoversion rate to 98% in vaccinated individuals. Immunoassays did not, however, reveal significant inter-group differences in seroconversion rates (≥95% in all groups). Log10 mean antibody neutralizing titers following the two-dose BBIBP-CorV protocol (IC50 = 2.21) were found to be significantly less than those succeeding moderate to severe COVID-19 (IC50 = 2.94). Prior SARS-CoV-2 positivity significantly increased post-vaccination antibody titers (IC50 = 2.82). Similar inter-group titer differences were obtained using the immunoassay. BBIBP-CorV post-vaccination titers may, thus, be lower than those following natural, moderate to severe infection, while prior SARS-CoV-2 exposure increases these titers to more closely approximate the latter.

## 1. Introduction

Since the start of the COVID-19 pandemic, there have been several troughs and peaks of the global infection rate. The frequency of new cases is declining currently, but WHO still considers the pandemic far from over [1]. As vaccination is one of the key strategies to ultimately halt the pandemic [2], questions related to vaccine immunogenicity and efficacy remain relevant. In many low and low–middle income countries, vaccination coverage is well below the target of 70% [3], a widely considered benchmark for herd immunity against COVID-19 [4]. None of the South Asian countries have achieved this target. In Pakistan, as of March 2022, only 45% of the population has been fully vaccinated with the two-dose vaccine protocol (Figure S1) [1,5].

Vaccines against COVID-19 aim to induce neutralizing antibodies against the spike (S) protein [6]. The presence and titer of neutralizing antibodies has established correlation to immunity against the infection, and neutralizing antibody assays detect inhibition of actual SARS-CoV-2 infection of target cells [7,8]. A modified version of the assay, using pseudotyped lentiviruses, increases the safety of the assay and facilitates detection [9]. This has been used to ascertain neutralizing antibody response to several viruses, including Ebola, MERS, and SARS [10]. On the other hand, immunoassays quantify antibodies binding to the S protein [11]. Though less challenging to perform, these assays exhibit variability in their potential to detect and quantify a neutralizing antibody response [12].

The vaccination campaign in Pakistan started in February 2021 [13]. Although seven vaccines are currently approved [14], BBIBP-CorV (Beijing Bio-Institute of Biological Products, Sinopharm, Beijing, China) was one of the first two vaccines to be authorized for emergency use in the country, and the first vaccine to be available for the COVID-19 vaccination campaign [15,16,17]. WHO approved a global BBIBP-CorV roll-out in May 2021 [18]. Although BBIBP-CorV is now approved for use in 90 countries (Figure S2), compared to some other vaccines, there is a paucity of published data reporting neutralizing antibody immune response following BBIBP-CorV in different populations [19]. We have not come across any published data from Pakistan which elucidates neutralizing antibody response in the population to any of the approved vaccines in the country.

**Figure 1:**
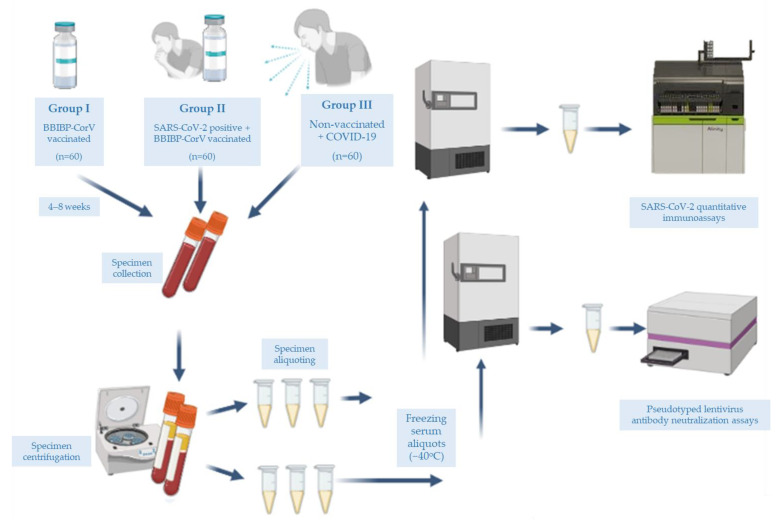
Study scheme showing the categorization of study groups, flow of specimens, and their storage until the final analysis by two different assays (image partly created in BioRender.com).

With regard to BBIBP-CorV administered to the Pakistani population, the questions we thus asked were: (1) what is the seroconversion rate following the two-dose BBIBP-CorV protocol; (2) how does the neutralizing antibody response of individuals vaccinated with two doses of BBIBP-CorV compare with that elicited following natural, moderate to severe COVID-19; and (3) can a widely used commercial immunoassay (SARS-CoV-2 IgG II Quant for use with Alinity i) provide information on the neutralizing antibody immune status of individuals following vaccination or COVID-19 positivity. The study scheme is depicted in Figure 1.

## 2. Materials and Methods

### 2.1. Participants and Specimen Collection 

In total, 180 participants were enrolled from 5th April to 24th August 2021, at the Indus Hospital and Health Network, Karachi, as shown in Figure 1. Group I vaccinated individuals did not have a history of positivity for SARS-CoV-2 or symptoms suggestive of COVID-19. Group II vaccinated individuals had prior SARS-CoV-2 positivity but with asymptomatic or mild disease. Group III unvaccinated individuals had one episode of moderate to severe COVID-19, requiring hospitalization. Clinical disease severity was ascertained according to the NIH guidance on COVID-19 disease severity [20].

Two tubes of serum specimens were collected from each participant, 4–8 weeks following either the second dose of vaccine in Group I and Group II, or onset of symptoms of COVID-19 in Group III. The mean interval between the second dose of vaccine and specimen collection was 36.6 ± 6.7 (SD) days and 38.2 ± 8.6 (SD) days for Groups I and II, respectively, while for Group III the mean interval between the onset of symptoms and specimen collection was 37.9 ± 7.5 (SD) (Table S1). The median intervals for the 3 groups were 36.5, 34.5, and 36.5, respectively.

### 2.2. Immunoassay

SARS-CoV-2 IgG II Quant immunoassay was performed on all specimens using Alinity i (Abbott), following the manufacturer’s instructions. Utilizing the chemiluminescent microparticle immunoassay (CMIA) technology, the assay quantifies immunoglobulin class G (IgG) antibodies to SARS-CoV-2. Briefly, the specimen and SARS-CoV-2 antigencoated paramagnetic microparticles are incubated together. Any SARS-CoV-2 IgG antibodies bind to the SARS-CoV-2 reagent antigen. Washing of this mixture is followed by the addition of anti-human IgG acridinium-labeled conjugate. The resulting luminescence is measured as relative light units (RLU) and reported as AU/mL. The amount of total IgG antibodies to SARS-CoV-2 are directly related to the RLU detected.

### 2.3. Cell Culture

HEK 293T cells (CRL-3216) were purchased from ATCC. HEK 293T cells expressing human ACE2 (#NR-52511) were provided by BEIResources, NIAID, NIH. Both cell types were cultured in complete medium comprising Dulbecco’s Modified Eagle’s Medium (DMEM, High Glucose; Gibco™ #11965092) supplemented with 10% Fetal Bovine Serum (Sigma-Aldrich # F9665), 1% Penicillin-Streptomycin (Gibco™ #15140122100), and 1% L-Glutamine (Gibco™ # G7513). Cell cultures were maintained in 5% CO_2_ at 37 °C in a humidified incubator. Cells used for the assays were all below passage eighteen.

### 2.4. Plasmids

Lentivirus vectors were packaged using the SARS-Related Coronavirus 2, Wuhan-Hu-1 Spike-Pseudotyped Lentiviral Kit V2 provided by BEIResources, NIAID, NIH (NR-53816). The kit is designed to generate pseudotyped lentiviral particles with the spike (S) glycoprotein gene. The backbone plasmid of the kit contains the luciferase (Luc2) and ZsGreen genes. Version 2 has the full-length S glycoprotein replaced with a C-terminally truncated spike. This is reported to increase lentivirus particle titers [21].

### 2.5. Lentivirus Preparation

To package lentiviruses, 5 × 10^6^ 293T cells were seeded per 10 cm tissue culture dish on day 1. Transformation of cells was undertaken 24 h later using BioT transfection reagent (# B01-01) and following plasmid DNA transfection protocol outlined in the manufacturer’s instructions. Before transformation, medium was changed to one without antibiotics (i.e., DMEM, High Glucose, 10%FBS, I% L-Glutamine). Following transformation, medium was changed 14–16 h later with one containing 5% FBS (i.e., DMEM, High Glucose, 5%FBS, 1% L-Glutamine, and 1% Penicillin-Streptomycin). Virus supernatant was harvested 24 h later, and fresh medium containing 5% FBS added. Virus supernatant was again harvested for two more consecutive days, filtered with 0.45 µm pore SFCA membrane, sterile filters (Corning ^®^ #431220, Arizona, USA), aliquoted, and frozen at −80 °C. Virus supernatant was prepared in one batch, of which a small aliquot was used to determine the lentivirus titer.

### 2.6. Lentivirus Titration

The 1 × 10^4^ HEK293T cells per well were seeded in a 96-well, flat bottom, tissue culture plate in 100ul complete medium. Then, 24 h later, an aliquot of lentivirus supernatant was thawed on ice, serially diluted 5-fold with complete medium seven times, and added to HEK293T cells, in a volume of 150 μL per well, after removing the complete medium. In total, 8 wells of a 96-well tissue culture plate were prepared in triplicate for the titration. Three extra wells were used to count the average number of cells per well. Medium was changed 24 h later, followed by incubation for another 24 h. Cells from each well were then harvested, washed thrice in PBS, and resuspended in 1ml PBS/well. Flow cytometric analysis for ZsGreen positivity was next performed on BD FACS™, and analysis completed using BD FACSDiva 8.0.2. (BD Biosciences, San Jose, CA, USA). Wells showing 1–10% positivity for ZsGreen were used for calculation of virus titer, expressed as Transduction Units (TU)/mL:

Titer (TU/mL) = (N × P)/(V × F)

N = Number of cells in each well, as averaged from 3 wells on the day of infection.

P = Percentage of cells positive for ZsGreen on flow cytometer.

V = Volume of virus (in mL) used for infection per well.

F = Fold dilution of virus in the selected well.

The virus titer used for the assays was 2.7 × 10^4^ ± 0.01 TU/mL.

### 2.7. Pseudotyped Lentivirus Antibody Neutralization Assay

The assay was performed as previously described with minor variations [22]. Briefly, 1 × 10^4^ 293T-ACE2 cells were seeded per well of a flat, clear-bottom, black-walled, poly-L-lysine treated plate (ThermoFisher Scientific™ #152037, Waltham, MA, USA). Then, 20–22 h later, five serum specimens were serially diluted in 1-5 dilutions in 150 μL of previously titrated virus in a 96-well, sterile, clear, flat bottom plate. Starting with a 1-5 dilution, each specimen was serially diluted 7 times and in duplicate. Serum and virus were subsequently incubated together for 1 h at 37 °C in a humidified incubator. Virus–serum mixtures were then added to 293T-ACE2 cells plated the previous day. Eight wells each of Virus Control (VC) and Cell Control (CC) were also plated (Figure S3).

VC comprised wells with 293T-ACE2 cells infected with 150μL of virus only without serum specimens, while CC constituted 293T-ACE2 cells with 150μL of complete medium only, without serum specimens or virus. Virus Control thus represented maximum possible infection of 293T-ACE2 cells, equivalent to what would be expected without the presence of any neutralizing antibodies in the serum specimens. Cell Control, on the other hand, represented maximum possible neutralization of the virus with antibodies. Medium was changed 24 h later, and the plate incubated for another 48 h. Assay readout was next performed by adding 30 μL of reconstituted luciferase reagent (Bright-Glo Luciferase Assay System, Promega # E2610, Madison, WI, USA) after removing 100 μL of medium. Luminescence was subsequently determined as RLU (Relative Luminescence Units) using a Varioskan™ LUX multimode microplate reader #VLBL00D2 (ThermoFisher Scientific, Waltham, MA, USA) and SkanIt™ Software for Microplate Readers (#5187149, ThermoFisher Scientific, Waltham, MA, USA).

### 2.8. Data Analysis

The average RLU from each specimen dilution was plotted, following normalization, with VC representing 100% infection and CC 0% infection, against the corresponding log10 dilution factor of each specimen. The half-maximal inhibitory concentrations (IC50) were calculated using a nonlinear regression algorithm (log[inhibitor] versus normalized response variable slope) in GraphPad Prism 9. IC50s values obtained from GraphPad Prism 9 were converted to a log10 scale. The mean coefficient of variation across duplicates was 0.12 ± 0.09 (SD). Similarly, immunoassay results were also log10 transformed. To ascertain the statistical significance of the mean log10 antibody titers between groups, the Wilcoxon rank-sum test was used. Results were considered statistically significant if the *p*-value obtained using the two-sided test was below 0.05. Pearson’s *r* was used to report correlations between log10 antibody titers obtained using SARS-CoV-2 IgG II Quant immunoassay and the log10 of IC50s of each specimen using the pseudotyped lentivirus antibody neutralization assay.

### 2.9. Validation of the Pseudotyped Lentivirus Antibody Neutralization Assay

As the backbone plasmid used for the pseudotyped lentivirus packaging contains ZsGreen and Luciferase genes, it was theoretically possible to use fluorescence and luminescence as assay endpoints. While a neutralizing antibody response could be observed with a fluorescent readout when using a fluorescent microscope and flow cytometer (Figure S4, Table S2), this was not detectable using the plate reader.

Before conducting the assay on the study specimens, we confirmed the validity of the assay using a commercial ACE-2 IgG antibody (Recombinant Human ACE-2 Fc Chimera Catalog Number: 10544-ZN (0.2 μM)). Starting with a concentration of 10^2^ ug/uL, 5-fold dilutions were created in duplicate in 8 consecutive wells, and the assay performed as described earlier (Figure S5). In addition, we confirmed that the assay does not give positive results with known negative specimens by performing the assay on three different serum specimens collected before the pandemic (Figure S6). We also determined that passage number of 293T-ACE2 cells did not significantly affect the antibody titer for up to eighteen passages (Figure S7).

## 3. Results and Discussion

### 3.1. Seroconversion Rate following Two-Dose BBIBP-CorV Protocol Is Lower than That following Moderate to Severe COVID-19

**Figure 2:**
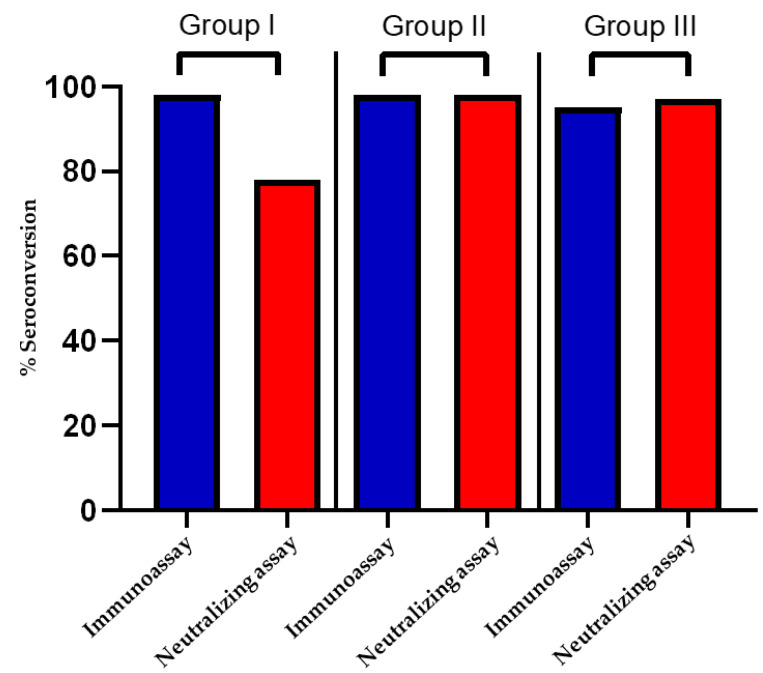
Seroconversion rate, using pseudotyped lentivirus antibody neutralization assay, and SARS-CoV-2 IgG II Quant immunoassay respectively, expressed as percentage of the total participants in each study group.

Figure 2 depicts the seroconversion rate in the three study groups as determined by the SARS-CoV-2 IgG II Quant immunoassay (Abbott), as well as the pseudotyped lentivirus antibody neutralization assay. The cutoff value for seroconversion was 50AU/mL for the immunoassay, while for the pseudotyped lentivirus antibody neutralization assay, an IC50 of 25 (1:25 serum dilution) was considered the cutoff [23]. Seroconversion rates, using neutralization assays, in vaccinated individuals (Group I) were lower (78%) than those in unvaccinated, COVID-19 recovered individuals (Group III) with moderate to severe infection (97%). Prior PCR positivity increased seroconversion rate to 98% in vaccinated individuals (Group II). Using the χ2 test of independence, the difference in seroconversion rates between Group I and Group II, as well as between Group I and Group III were found to be statistically significant (χ2 *p*-value < 0.05 for both comparisons). Immunoassays did not, however, reveal significant inter-group differences in seroconversion rates (≥95% in all groups, χ2 *p*-value > 0.05 for all inter-group comparisons).

Using antibody neutralization assays, a phase I/II trial evaluating the safety and immunogenicity of BBIBP-CorV vaccine has reported this seroconversion rate as 100%, at four weeks following the second dose of the vaccine in participants aged 18–59, and at 42 days in participants more than 60 years old [24]. The study, however, reports seroconversion as an increase in the neutralization antibody titer over baseline, determined prior to vaccine administration. Using an IC50 cutoff of 4 (1:4 serum dilution) [25], Zhang et al. reported 90.7% seropositivity following two doses of BBIBP-CorV. Li et al. report a seroconversion rate of 73.97% at 8 weeks post second BBIBP-CorV dose [26].

Other studies have used immunoassays and ELISA to determine seroconversion rates following BBIBP-CorV administration. Petrović et al. used the quantitative LIAISON SARS-CoV-2 S1/S2 IgG assay and reported a seroconversion rate of 83% [27]. Jeewandara et al. used ELISA and described 60.9% seropositivity with SARS-CoV-2 specific antibodies against the ACE2 receptor [28]. Fu et al. report 75% seroconversion rate using SARS-CoV-2 RBD ELISA [29], while Elgendy et al. reveal 49% seroconversion, but have tested participants at Day 18 following BBIP-CorV vaccination [30,31]. Alqasseih et al. report 85.7% seroconversion at 6 weeks post two-dose BBIBP-CorV vaccine schedule, using a qualitative immunoassay [32], while Lijeskić et al. [33] have reported 81.7% seroconversion, 6 weeks post-vaccination, using a two-step sandwich enzyme immunoassay method for RBD anti-S antibodies.

Variation across studies in the assays employed, methodologies used to report results, populations studied, as well as the specific time points of specimen collection are factors possibly accounting for variation in the reported seroconversion rates following administration of the BBIBP-CorV. As a case in point, the immunoassay used in our study quantifies total IgG antibodies, unlike the neutralizing assay which detects only neutralizing antibodies, and has thus shown high seroconversion rates when compared to the latter. However, since neutralizing antibodies are more strongly correlated to actual vaccine efficacy, results from the neutralizing assay may be more representative of the actual immunogenicity following vaccine administration. It must be highlighted though that most studies, including the present one, report on seroconversion rates relatively early on following vaccination, while several studies have shown a sharp decline in antibody titers after a few months following vaccination [28].

### 3.2. Mean Antibody Titers following Two-Dose BBIBP-CorV Protocol Are Lower than That following Moderate to Severe COVID-19

**Figure 3:**
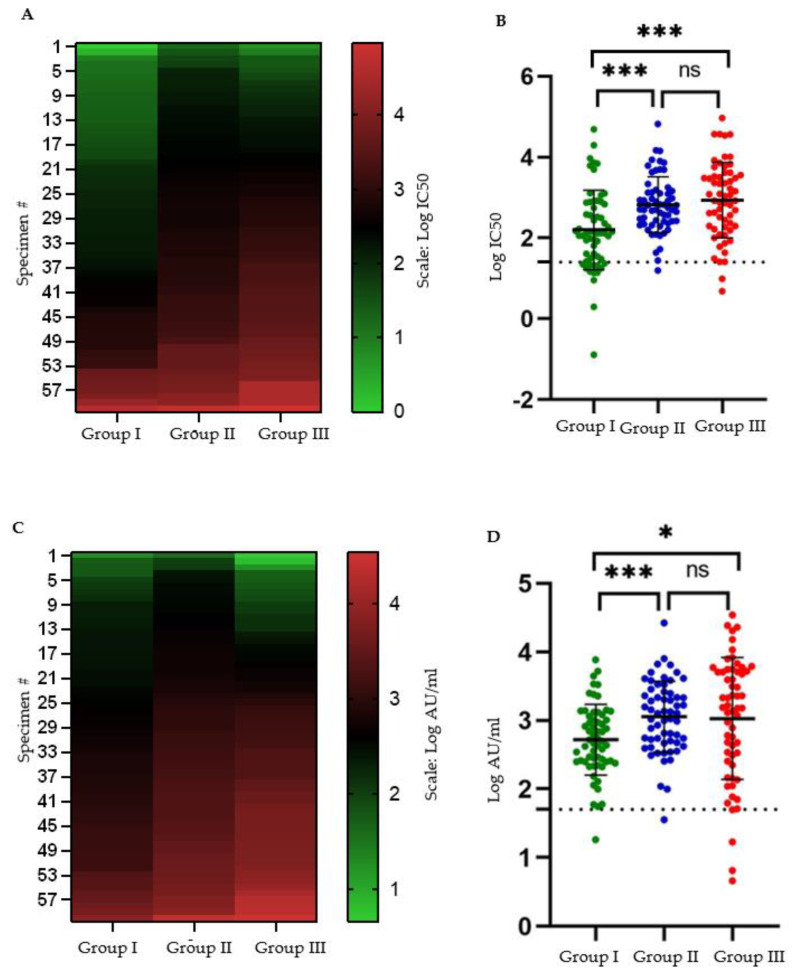
Log10 IC50 (**A**,**B**) and log10 AU/mL (**C**,**D**) values obtained following pseudotyped lentivirus antibody neutralization assay, and SARS-CoV-2 IgG II Quant immunoassay, respectively, on specimens from each of the three groups of study participants. The horizontal dotted lines in B and D indicate the assay cutoff at IC50 = log10(25) for the neutralization assay, and Log10(50) AU/mL for the immunoassay, respectively. # = number, ns: not significant, *: *p* < 0.05, ***: *p* < 0.005. (Note: values below zero have been rounded to zero in panels **A** and **C**).

BBIBP-CorV vaccinated individuals had a significantly lower log10 mean antibody titer (immunoassay: 2.72 AU/mL; neutralizing assay IC50: 2.21) than that produced by moderate to severe COVID-19 (immunoassay: 3.06 AU/mL; neutralizing assay IC50: 2.94). Prior SARS-CoV-2 positivity increased the log10 mean post-vaccination titers (immunoassay: 3.03 AU/mL; neutralizing assay IC50: 2.82) (Figure 3). Other studies undertaken on Argentinian and Egyptian health care workers, respectively [34,35], and the general population in Serbia [28] have also documented prior SARS-CoV-2 exposure increasing BBIBP-CorV post-vaccination antibody titers. Meanwhile, a few studies have also shown that antibody titers following natural infection in unvaccinated individuals are higher than those following BBIBP-CorV vaccination [27,33,36,37].

Most of these studies, however, have not segregated participants by disease severity. We additionally show that mean antibody titers, including neutralizing antibodies, produced following one moderate to severe COVID-19 episode, are almost equivalent to those following the two-dose BBIBP CorV protocol with additional prior exposure to SARS-CoV-2 in the form of mild or asymptomatic infection.

Studies comparing immunogenicity of mRNA vaccines to that of BBIBP-CorV have generally reported lower seroconversion rates and antibody titers in the latter. Lijeskić et al. [33] investigated three vaccines: BNT162b2, BBIBP-CorV, and Gam-COVID-Vac, and observed less potent immune responses in BBIBP-CorV as compared to the other two, while COVID-19 convalescents in this study demonstrated higher titers than BBIBP-CorV, but less than BNT162b2. Petrović et al. [27] similarly reported lower seroconversion rates and antibody titers following BBIBP-CorV than either BNT162b2 or Gam-COVID-Vac.

### 3.3. There Is a Moderate Correlation between Antibody Titers Obtained via the Pseudotyped Lentivirus Antibody Neutralization Assay and the SARS-CoV-2 Quant II Immunoassay (Abbott)

**Figure 4:**
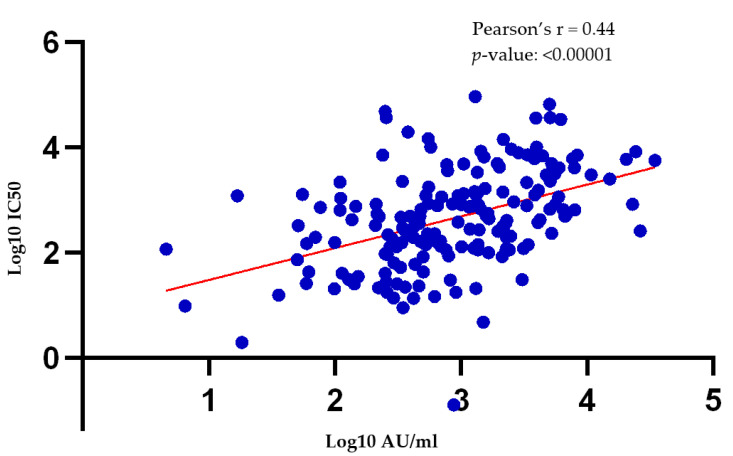
Correlation of log10 IC50 and log10 AU/ml values obtained following pseudotyped lentivirus antibody neutralization assay, and SARS-CoV-2 IgG II Quant immunoassay, on samples from each of the three groups of study participants.

There is a moderate correlation between the antibody titers obtained using the pseudotyped lentivirus antibody neutralization assay, and SARS-CoV-2 IgG II Quant immunoassay (Figure 4, Figure S8). SARS-CoV-2 IgG II Quant immunoassay detects all IgG antibodies against SARS-CoV-2, which includes neutralizing antibodies against the receptor binding domain (RBD) of the SARS-CoV-2 spike (S) protein, but are not exclusive to it. Chapuy-Regaud et al. also report a moderate correlation (Spearman’s *r* = 0.63) between the results of SARS-CoV-2 IgG Quant immunoassay and live virus neutralization assays in known positive samples [38].

SARS-CoV-2 IgG II Quant immunoassay, thus, gave similar differences in mean antibody titers between study groups (Figure 3) as those generated using the pseudotyped lentivirus antibody neutralization assay. For large-scale studies intending to discover inter-group/population differences, the test may be a useful surrogate for the neutralizing antibody assays as the latter are more cumbersome to perform and require specialized technical expertise. Results of individual tests using the SARS-CoV-2 IgG II Quant immunoassay, however, need to be interpreted with caution, and in accordance with the tests’ intended use, i.e., measurement of total SARS-CoV-2 IgG antibodies rather than only neutralizing ones.

### 3.4. No Significant Differences in the Mean Antibody Titers of the Study Participants Were Observed Based on Their Sex or Age Bracket

**Figure 5:**
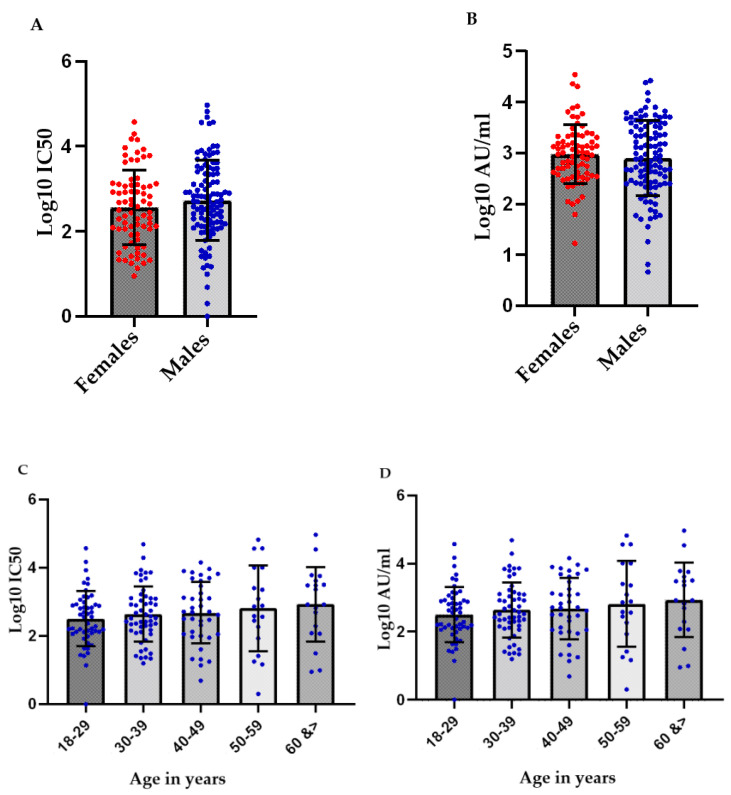
Distribution of log10 IC50 and log10 AU/ml values obtained following pseudotyped lentivirus antibody neutralization assay, and SARS-CoV-2 IgG II Quant immunoassay by sex (**A**,**B**) and age (**C**,**D**).

No significant differences in the log10 mean antibody titers were observed based on the sex or age of the participants (Figure 5). Overall, though these titers appear to increase slightly with age, the difference was not statistically significant, and could be explained by a relatively greater representation of Group III participants in the higher age brackets (Table 1). Lack of correlation of age with antibody titers post-BBIBP CorV has been shown by Petrović et al. [27], and El Ghitany et al. [35] as well, though Ma et al. [36] and Ferenci et al. [19] have reported a negative correlation of post-vaccination immunity and age. Zhang et al. also show a small negative correlation with age (r = −0.36), while age was non-significant in the female sub-group of those study participants [26]. All these studies report no sex-based differences in the antibody titers following BBIBP CorV vaccination.

Our results, thus, match other previously conducted ones with regard to a lack of sex-based differences in antibody titers post vaccination. Data, however, are conflicting with regard to variation in the titers with age. Methodological variations, age groups included in the study, as well as sample size differences are likely to contribute to variability of reported results. In the present study, age matching, as well as a larger sample size, could bring out some differences in the antibody titers based on the age. However, these differences are unlikely to be marked as even the studies describing such a difference report only a mild negative correlation of antibody titers with age.

Moreover, segregating the results by the study groups (Figure S9) showed that antibody titer correlation with age decreased within the vaccinated group, just as reported in the previously mentioned studies, but prior SARS-CoV-2 infection or COVID-19 infection in naïve individuals had a positive correlation of age with immunity. Such a pattern of correlation of immunity with age following natural infection and immunity is also reported by Petrović et al. [27]. Notwithstanding, all correlations observed in the present study with age were weak and not statistically significant.

Petrović et al. [27] and Lijeskić et al. [33] have compared the correlation of BNT-162b2 and BBIBP-CorV with age in the same studies. Both of these studies reported weak and/or statistically insignificant correlations of antibody titers following either of these vaccines and ages of study participants.

In conclusion, immune response to BBIBP-CorV, with or without prior SARS-CoV-2 exposure, and that following moderate to severe COVID-19 has no sex-based differences. Any correlation with age, if present, is weak but could have become statistically significant had the sample size been higher.

### 3.5. The Interval between Sample Collection and the Second Vaccine Dose (Group I and Group II)/Onset of Symptoms (Group III) Had No Correlation with Antibody Titers

The interval, in days, between the second vaccine dose (Group I and Group II)/onset of symptoms (Group III) and sample collection was restricted to 4–8 weeks. The time periods were selected based on published data indicating that neutralizing antibody titers peak between 4 and 8 weeks following onset of symptoms of COVID-19 [39,40,41], or vaccination [24,42], and remain fairly constant throughout this period. Applying Pearson’s *r*, we did not observe statistically significant correlations between the sample collection intervals and the respective antibody titers with both the immunoassay and the neutralizing assay (*p*-values > 0.05 for all comparisons).

Feng et al. [39] have performed longitudinal profiling of antibody response in COVID-19 recovered individuals and observed the antibody titers to peak between 4 and 8 weeks days post onset of COVID-19 symptoms. Longueira et al. [40] have similarly determined IgM and IgG antibody titers following onset of COVID-19 symptoms until 12 weeks, and have shown the levels to peak at 5 weeks and to plateau until 7 weeks. Glück et al. [41] also show stable IgG titers between 3 and 12 weeks post onset of COVID-19 symptoms.

### 3.6. Limitations

To our knowledge, this is the first published study using pseudotyped lentivirus neutralizing antibody assays on a sample of the Pakistani population to ascertain immunogenicity of a vaccine included in the government vaccination campaign, and administered to a large percentage of the population. The study, however, did have some limitations.

The main limitations of this study are a relatively small sample size, and a lack of matching for age and sex between groups. The study also did not assess immunogenicity following naïve, mild COVID-19, inclusion of which as a separate study group, would have allowed the comparison of immunogenicity of BBIBP-CorV with mild infection. Given that we did not have the resources to further increase the sample size, this would, however, have reduced the sample size per group. For the same reason, we did not assess if prior moderate to severe COVID-19 boosts the immune response following subsequent vaccination, though we cannot surmise a reason for not expecting an immune response of at least the same magnitude as that following mild/asymptomatic infection and vaccination.

We did not measure antibody titers of participants before enrollment in the study. Since the infection was widespread by the time the specimens were collected, we cannot entirely be certain if Group I participants were not exposed to asymptomatic SARS-CoV-2 in the past. Ruling out such an exposure could have magnified the difference observed between the Group I and the other two study groups. Further, in individuals showing low antibody titers or lack of seroconversion, a T cell immune response cannot be ruled out. Seroconversion rates and antibody titers, thus, must be interpreted with caution, as they are not sole indicators of vaccine efficacy [27]. Moreover, data were generated from a limited timeframe following infection, and further studies evaluating long-term immunogenicity of the vaccine in the Pakistani population are needed. The latter is also important because antibody titers are reported to decrease with time following BBIBP-CorV administration [28].

The study assessed response following a two-dose vaccine protocol, while at the time of writing booster vaccines are also available to the general population in Pakistan. At the time of specimen collection, however, booster vaccines were not yet available. In addition, even today, less than half the population in Pakistan is vaccinated with the two-dose vaccine protocol, implying that study of immunogenicity following the two-dose protocol is still relevant for most of the Pakistani population. Notwithstanding, in a small, recent study by Zhang et al., a homologous booster dose with BBIBP-CorV has shown to increase mean neutralizing antibody titers beyond the peak produced by the two-dose regimen [43]. Cheng et al. report these titers to be 13.2 times the mean pre-booster level [44] while Ai et al. report these titers to be up to 15-fold higher than baseline [45].

The various spike protein variants found in populations across the globe were not assessed in the study. Study of immunity to SARS-CoV-2 S variants is, and will continue to be, a moving target as new variants will continue to arise. Regardless, in the absence of established genomic surveillance mechanisms in the country, data on the prevalence and types of SARS-CoV-2 mutations in Pakistan has remained sparse at all times. Subsequently arising variants have shown reduced titers to vaccines, and it is thus entirely possible that testing for antibody titers with these spike protein mutations might have further reduced the detected antibody titers in Group I [46,47,48,49] and magnified the inter-group difference.

We also did not have data on the variants which the study participants in Group III were infected with. Globally, the Delta variant was the predominant one during the specimen collection period, but data on the prevalence of the various SARS-CoV-2 variants from Pakistan was sparse to absent. Assuming the mutation prevalence in Pakistan reflected the general global trend at that time, testing for immunogenicity against the Delta S protein mutations would again have magnified the difference observed between Group I and Group III as Delta variants also have been reported to reduce neutralizing antibody titers post vaccination [50,51,52].

## 4. Conclusions

Immunogenicity of the two-dose BBIBP-CorV protocol may be less than that of moderate to severe COVID-19. Prior SARS-CoV-2 positivity enhances this immunogenicity to about the same level as that following moderate to severe natural infection. This has implications for the need of a booster dose in people vaccinated with a two-dose protocol of BBIBP-CorV, which may bring the neutralizing antibody titer closer to that produced by natural, moderate to severe infection. For further studies assessing neutralizing antibody response to vaccines or natural infection, Abbott Alinity i SARS-CoV-2 Quant Immunoassay could be an acceptable alternative to the neutralizing antibody assays for detecting, large scale, inter-group differences, but may not be ideal to document individual neutralizing antibody titers.

## Figures and Tables

**Table 1:** Age and sex distributions of study participants.

	Sex		Age Brackets (Years)				
	F	M	18–29	30–39	40–49	50–59	60 & >
Group I	26	34	12	23	16	8	1
Group II	29	31	24	20	15	1	0
Group III	19	41	15	10	7	10	18
Total	74	106	51	53	38	19	19

## Data Availability

Data are contained within this article and the associated Supplementary Materials.

## Supplementary Materials

The following supporting information can be downloaded at: https://www.mdpi.com/2076-393X/10/5/692/s1.
