# Supplementary material for: Neutralizing Antibody Response to BBIBP-CorV in Comparison with COVID-19 Recovered, Unvaccinated Individuals in a Sample of the Pakistani Population
Source: Vaccines (Basel). 2022 Apr 28;10(5):692. doi: 10.3390/vaccines10050692 (PMC9171576; doi:10.3390/vaccines10050692)
Supplement: Supplementary file 1 [file vaccines-10-00692-s001.zip › vaccines-1672058-supplementary.pdf]

vaccines

Supplementary Figures and Tables

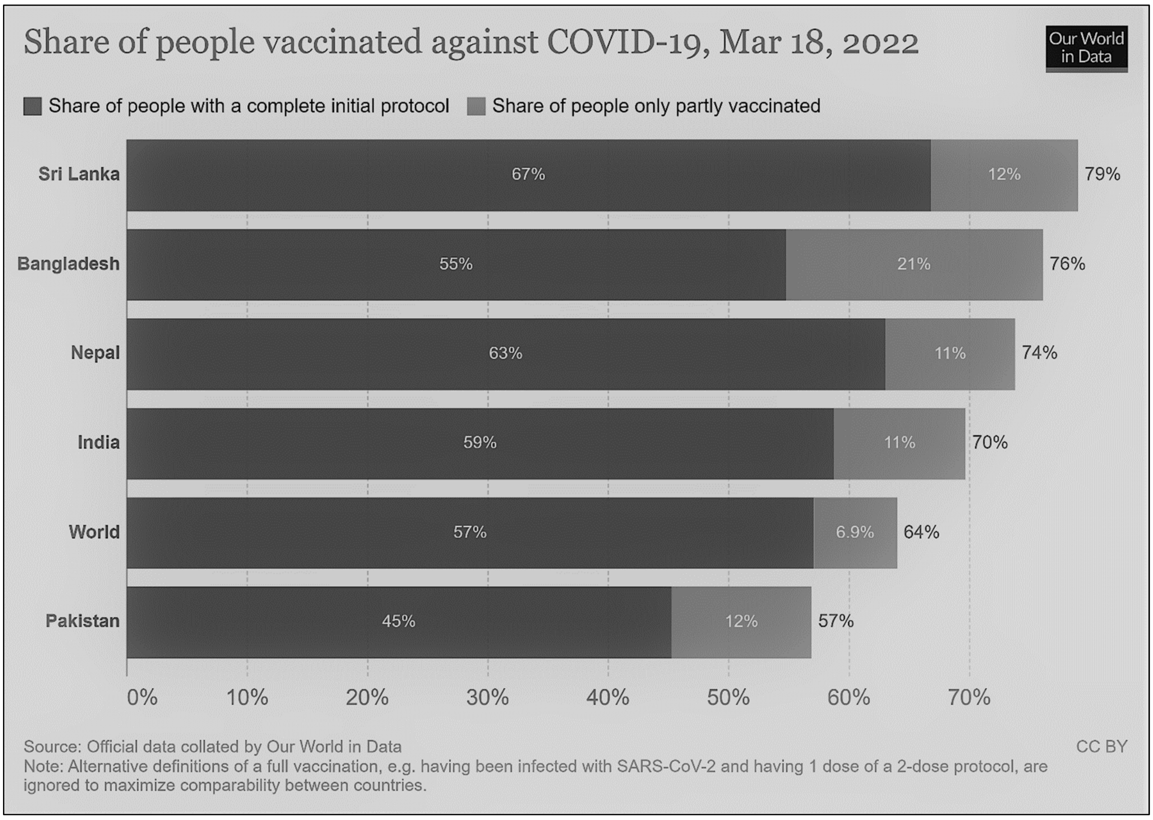

Figure S1. Percentage of people vaccinated against COVID-19 in South Asia in comparison with the world average (Hannah Ritchie, Edouard Mathieu, Lucas Rodés-Guirao, Cameron Appel, Charlie Giattino, Esteban Ortiz-Ospina, Joe Hasell, Bobbie Macdonald, Diana Beltekian and Max Roser (2020) - "Coronavirus Pandemic (COVID-19)". Published online at OurWorldInData.org. Retrieved from: 'https://ourworldindata.org/coronavirus'. (Accessed on 18 March 2022).

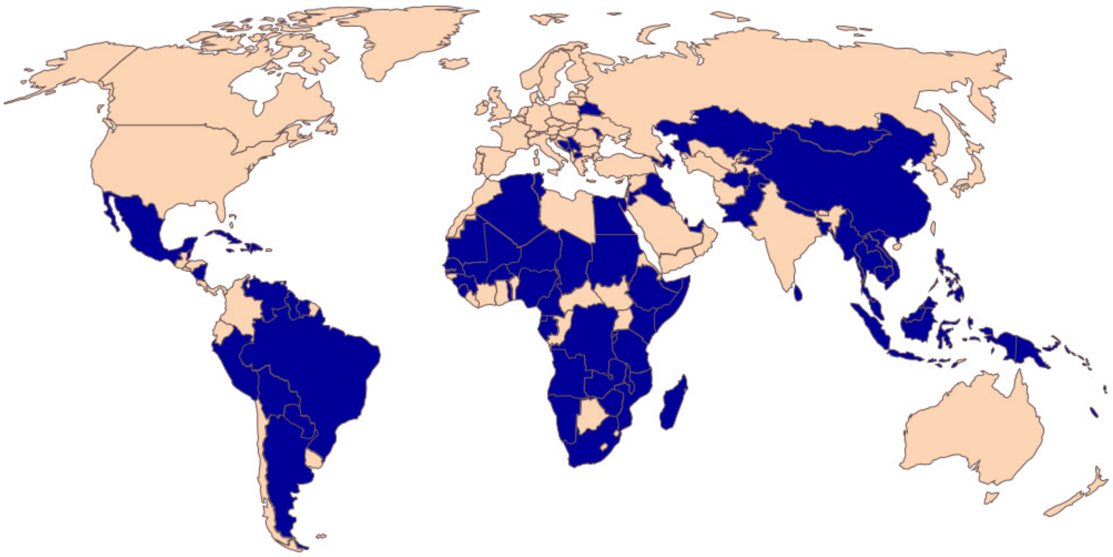

Figure S2. World map with countries where BBIB-CorV is approved for use (blue). Source: www.freeworldmaps.net (Accessed on 15 March 2022).

Table S1. Raw data per specimen segregated according to the three study groups, and including the interval in days between the second dose of vaccine and specimen collection for Group I and Group III, and between symptom onset and specimen collection for Group III (second column from the left); results of the Abbott SARS-CoV-2 Quant II immunoassay in AU / ml converted into their respective log10 values (third column from the left); and log 10 IC50 of the pseudotyped lentivirus antibody neutralization assays (last column from the left).

Specimen #	Days post 2nd vaccine dose/symptom onset	Log AU / ml (immunoassay)	Log IC50 (neutralizing assay)
		Group I	
1	31	3.4	2.1
2	38	3.1	2.2
3	37	3.9	3.8
4	39	2.4	2.0
5	32	3.7	3.7
6	33	2.6	2.5
7	54	3.1	2.9
8	33	2.4	2.0
9	31	2.6	1.1
10	31	2.5	2.3
11	33	3.1	2.4
12	56	3.1	2.9
13	32	1.3	0.3
14	33	3.1	1.3
15	48	2.9	1.5
16	32	2.0	1.3
17	32	2.8	3.1
18	31	2.3	2.9
19	31	2.6	4.3
20	36	2.3	2.5
21	32	2.5	1.0
22	32	2.7	2.4
23	37	2.6	2.6
24	36	2.7	1.4
25	38	2.6	1.3
26	33	2.4	1.6
27	33	2.1	1.5
28	51	3.1	3.1
29	33	3.0	1.3
30	33	2.9	1.9
31	52	2.8	2.1
32	37	3.0	2.6
33	33	1.8	1.4
34	34	2.7	2.2
35	34	2.4	1.3
36	31	2.4	3.9
37	31	3.4	2.1
38	52	2.5	1.1
39	32	2.8	2.2
40	33	3.0	2.1
41	30	2.2	1.5
42	39	3.0	2.1

43	31	1.8	2.2
44	32	3.1	2.9
45	33	2.3	1.3
46	47	3.6	3.8
47	35	2.3	2.7
48	35	3.5	2.2
49	39	2.8	1.2
50	31	3.2	2.8
51	53	2.4	4.7
52	35	3.4	4.0
53	40	2.9	-0.9
54	35	2.1	1.6
55	31	2.5	1.8
56	37	3.5	2.9
57	32	2.4	2.1
58	48	1.7	3.1
59	41	2.7	1.9
60	39	2.9	2.1

		**Group II**	
**1**	**29**	**3.7**	4.8
2	33	3.4	2.3
3	31	3.5	3.9
4	32	3.3	2.4
5	31	2.5	2.5
6	30	3.3	4.2
7	32	2.8	2.4
8	30	3.8	2.7
9	31	3.1	3.2
10	54	3.6	3.1
11	57	3.4	3.0
12	44	3.2	2.7
13	35	3.4	2.1
14	43	3.6	3.8
15	53	2.9	3.7
16	35	2.7	2.7
17	55	3.3	2.4
18	34	3.9	2.8
19	29	4.4	2.4
20	32	2.5	1.7
21	47	3.5	3.9
22	33	3.1	3.1
23	54	2.5	2.7
24	45	2.8	2.2
25	34	3.5	3.3
26	40	2.8	2.9
27	50	2.7	2.9
28	33	2.6	2.4
29	33	2.6	2.7

30	29	2.4	2.3
31	34	3.6	2.6
32	35	3.0	2.9
33	33	3.2	2.7
34	31	3.1	2.1
35	31	3.3	3.6
36	29	2.7	3.2
37	40	3.6	3.2
38	33	2.6	2.3
39	31	3.6	2.6
40	30	2.0	2.2
41	38	3.2	3.9
42	31	3.2	2.6
43	38	2.4	1.4
44	56	3.0	3.0
45	28	2.9	2.9
46	53	2.7	3.1
47	51	3.0	3.1
48	37	2.7	4.2
49	50	3.3	3.7
50	48	3.8	2.8
51	38	1.6	1.2
52	54	3.1	2.1
53	39	2.7	2.6
54	41	2.6	2.3
55	37	2.7	1.6
56	34	3.7	2.8
57	33	3.0	3.7
58	40	2.0	2.8
59	32	2.5	2.1
60	37	3.3	2.5

**Group III**			
**1**	**28**	**3.2**	2.0
2	36	3.0	3.1
3	47	2.6	1.8
4	36	1.7	1.9
5	48	2.8	2.3
6	43	2.2	1.4
7	33	3.3	3.2
8	49	4.0	3.5
9	53	3.7	2.4
10	28	3.2	3.2
11	33	2.8	4.0
12	41	1.9	2.9
13	52	2.9	3.6
14	28	3.1	3.5
15	33	2.5	2.2
16	29	1.7	2.5

17	44	3.6	4.6
18	41	3.6	4.0
19	41	4.2	3.4
20	30	3.1	2.4
21	42	3.2	0.7
22	49	4.5	3.8
23	28	3.7	2.9
24	43	3.7	4.6
25	35	4.4	2.9
26	42	3.8	3.6
27	31	3.4	2.6
28	28	2.2	2.9
29	36	3.8	2.7
30	44	4.3	3.8
31	35	3.3	1.9
32	42	3.5	1.5
33	41	4.4	3.9
34	30	0.7	2.1
35	33	3.4	2.6
36	38	3.9	3.9
37	38	2.7	2.2
38	37	3.4	2.3
39	35	3.5	2.1
40	36	2.5	1.4
41	39	2.4	4.6
42	34	3.9	3.6
43	51	3.8	4.5
44	31	2.3	2.7
45	32	1.8	2.3
46	53	3.7	3.4
47	31	3.7	3.5
48	52	0.8	1.0
49	37	2.1	2.6
50	42	3.7	3.5
51	31	3.1	5.0
52	30	1.2	3.1
53	30	2.0	3.0
54	31	2.5	3.4
55	37	3.2	3.8
56	50	2.0	3.3
57	48	3.8	3.1
58	38	3.7	3.5
59	35	1.8	1.6
60	28	2.7	2.8

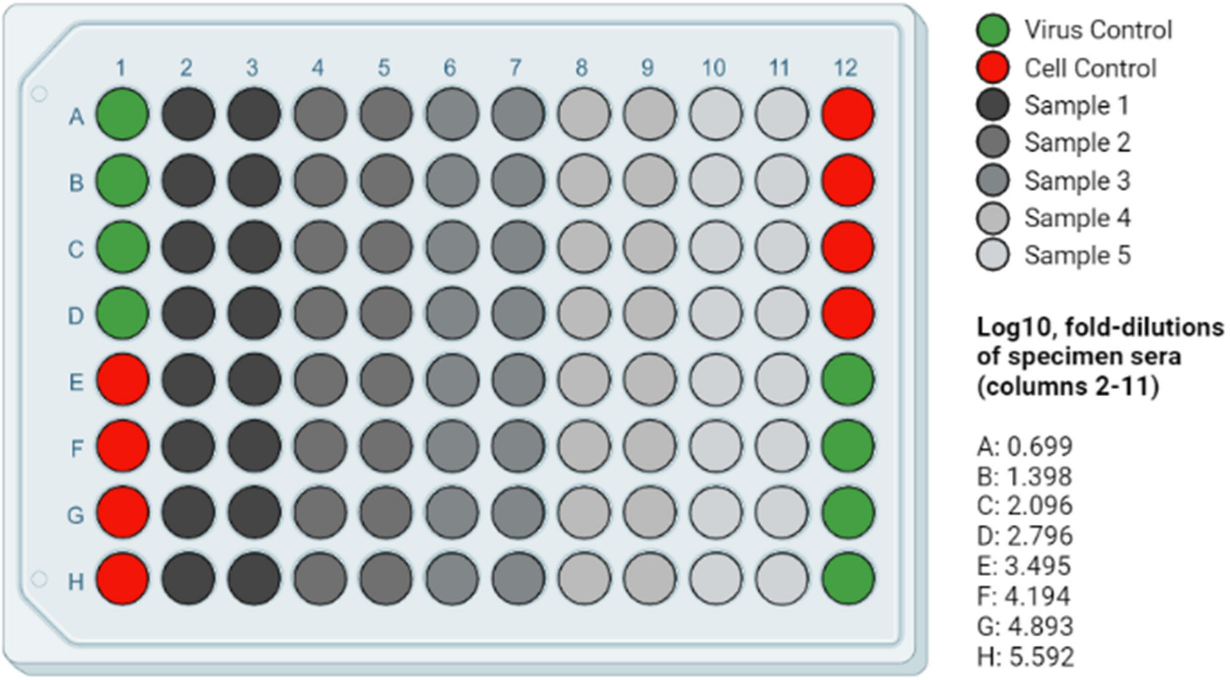

Figure S3. Plate map of black-walled, clear-bottom 96-well plates used for the final readout of the pseudotyped lentivirus antibody neutralization assay. Specimens, run in duplicates and shown in greyscale, were serially diluted vertically, starting from row A and ending at row H. The figure legend indicates the final row-wise fold-dilutions of sera used for each specimen, expressed as their corresponding log10 values (image created in BioRender.com).

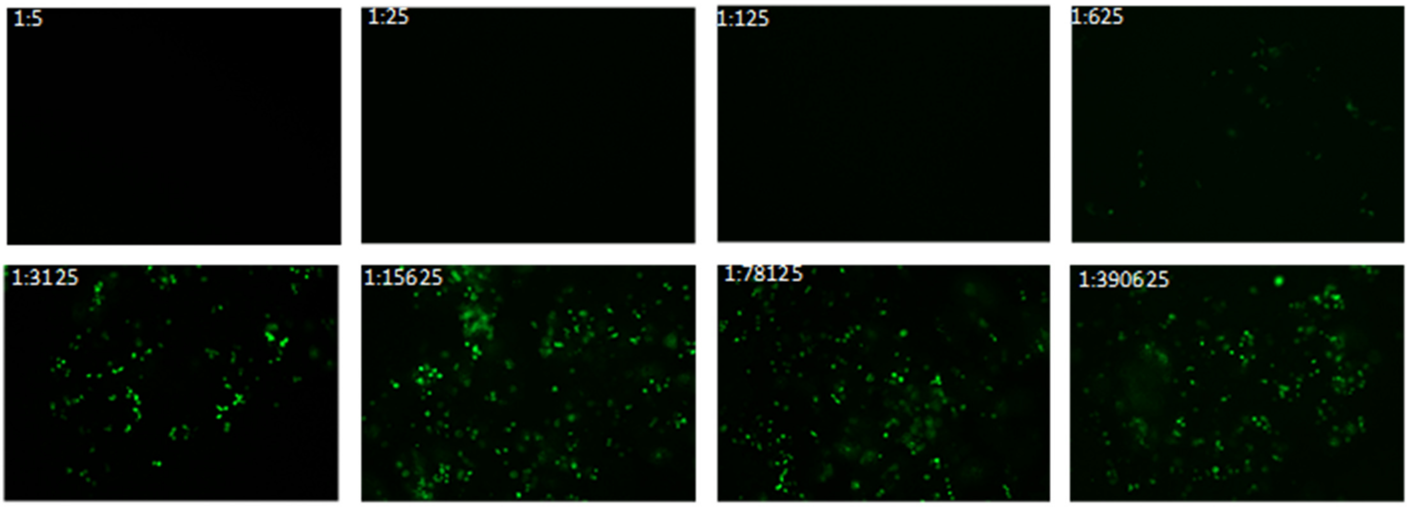

Figure S4. Fluorescent microscopic images of pseudotyped lentivirus antibody neutralization assay. The top left corner of each image shows the serum sample dilution used in the corresponding well.

Table S2. Serum sample dilution versus ZsGreen positivity on flow cytometry.

Serum dilution	ZsGreen % positivity
1:5	2.1
1:25	5.1
1:125	6.3
1:625	14.3
1:3125	16.5
1:15625	20.5
1:71825	27.2

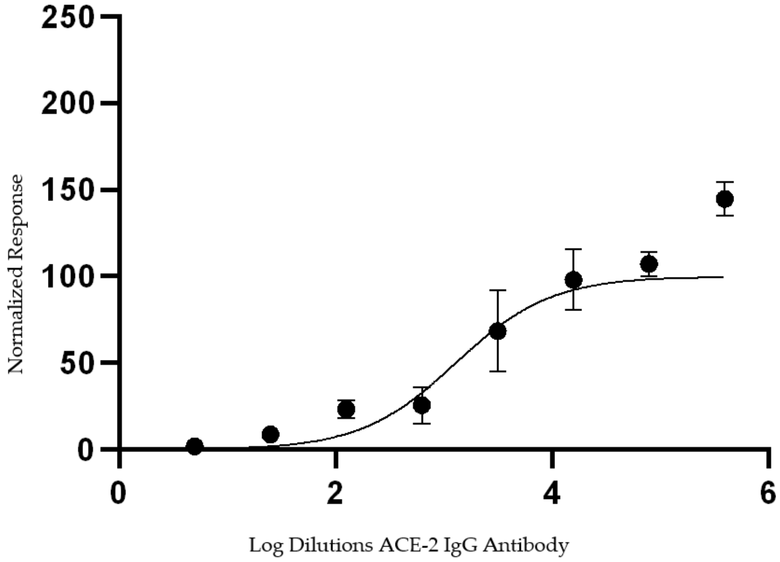

Figure S5. Log dilutions of ACE-2 IgG Antibody versus RLU expressed as normalized response.

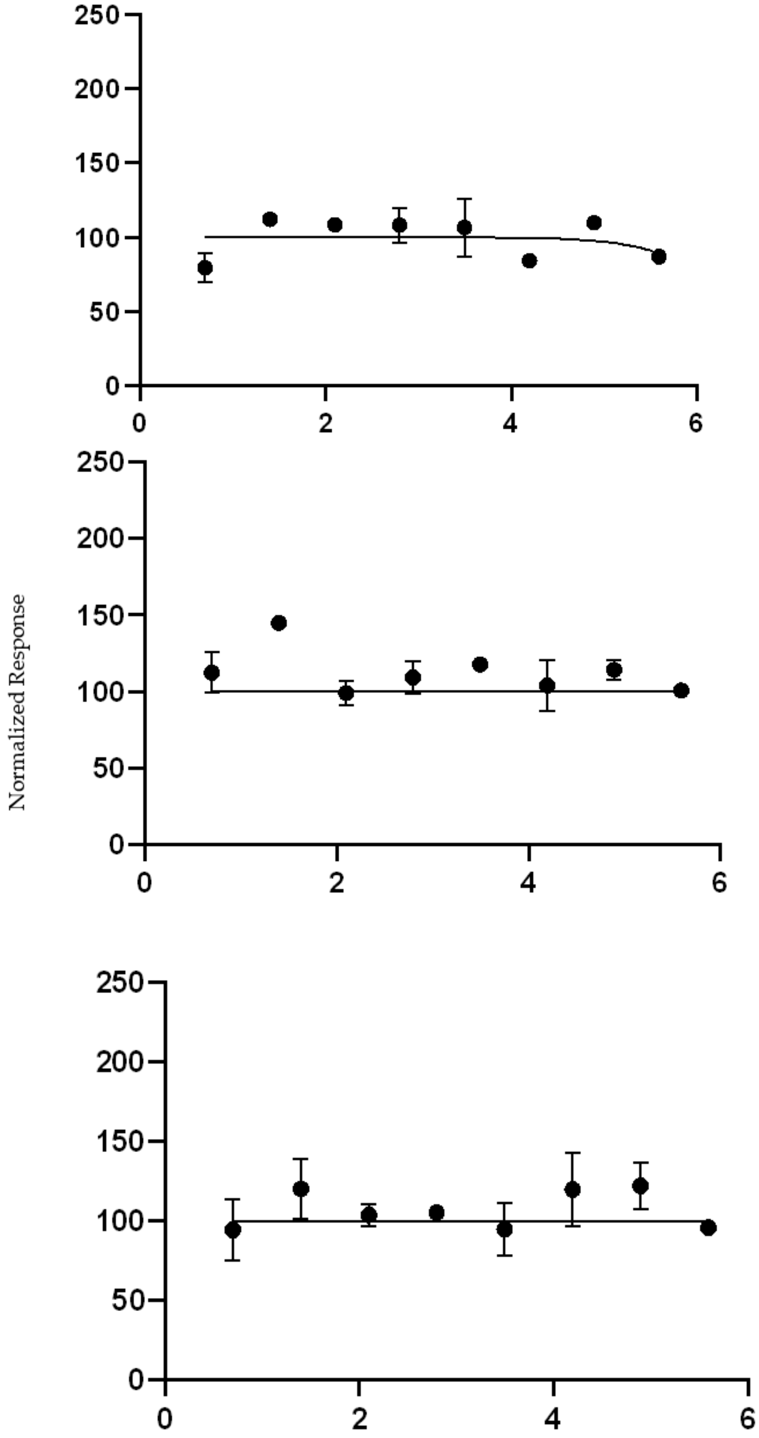

Figure S6. Log dilutions of pre-COVID-19 serum samples versus RLU expressed as normalized response.

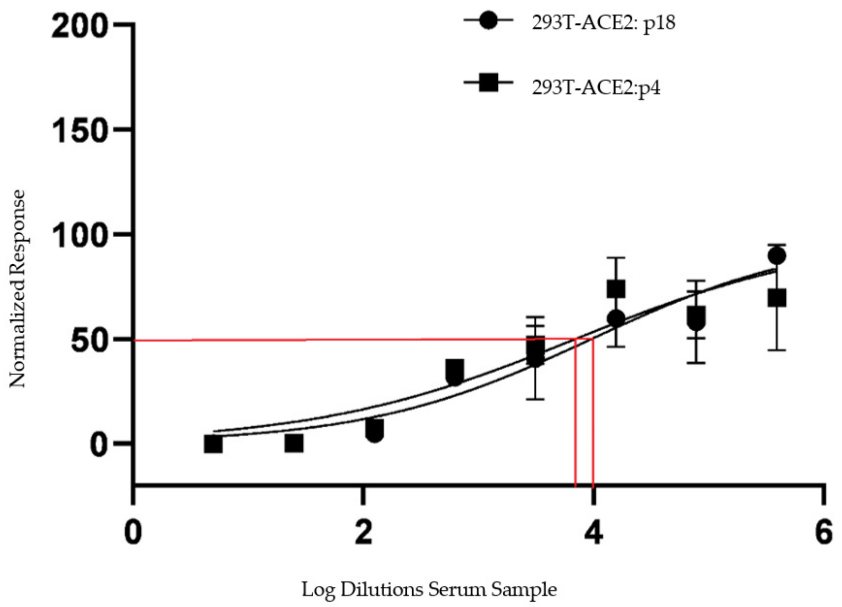

Figure S7. Log dilutions of serum samples versus RLU expressed as normalized response using 293T-ACE2 cells passage #4 versus passage #18.

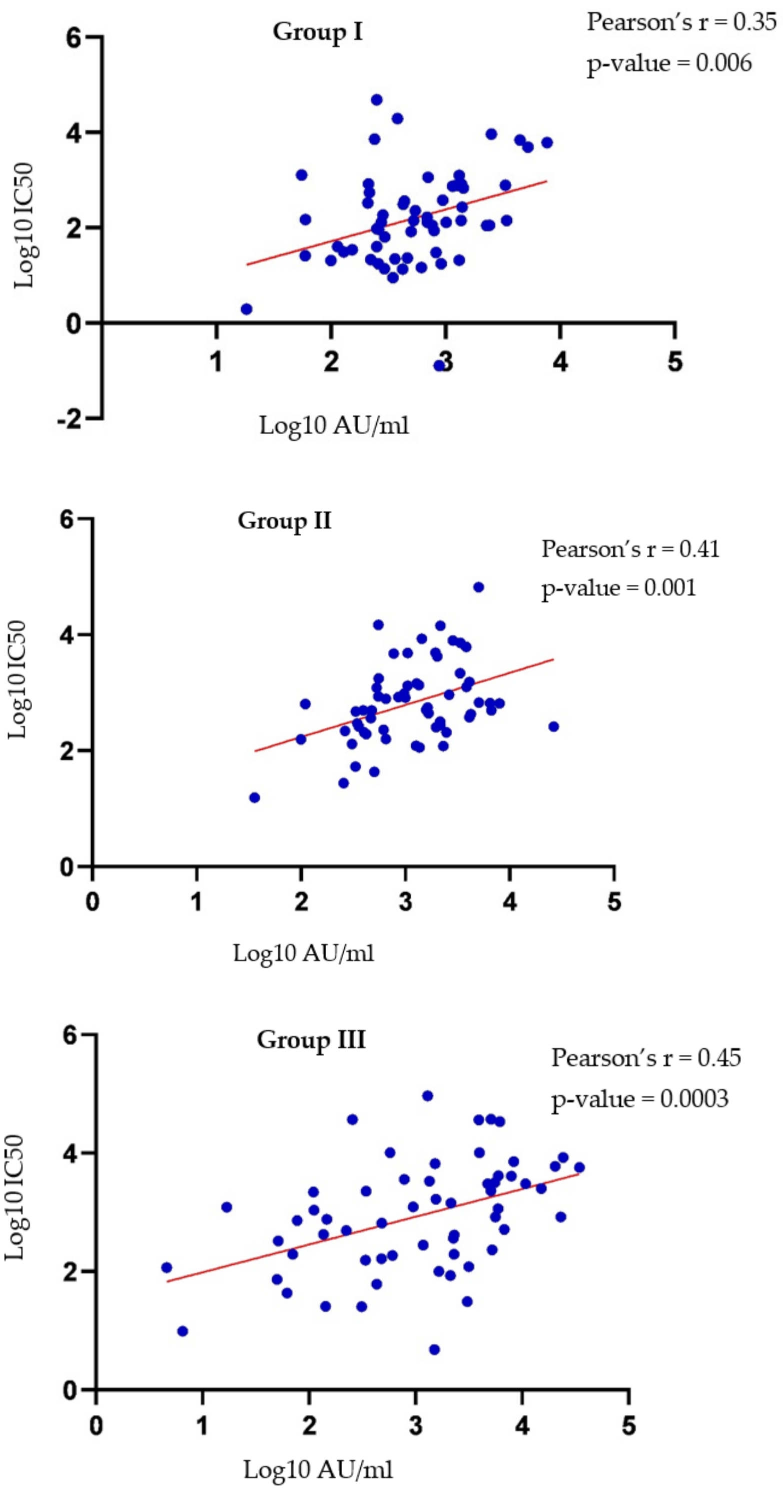

Figure S8. Correlation of log10 IC50 and log10 AU/mlvalues obtained following pseudotyped lentivirus antibody neutralization assay, and SARS-CoV-2 IgG II Quant immunoassay, segregated by the three groups of study participants.

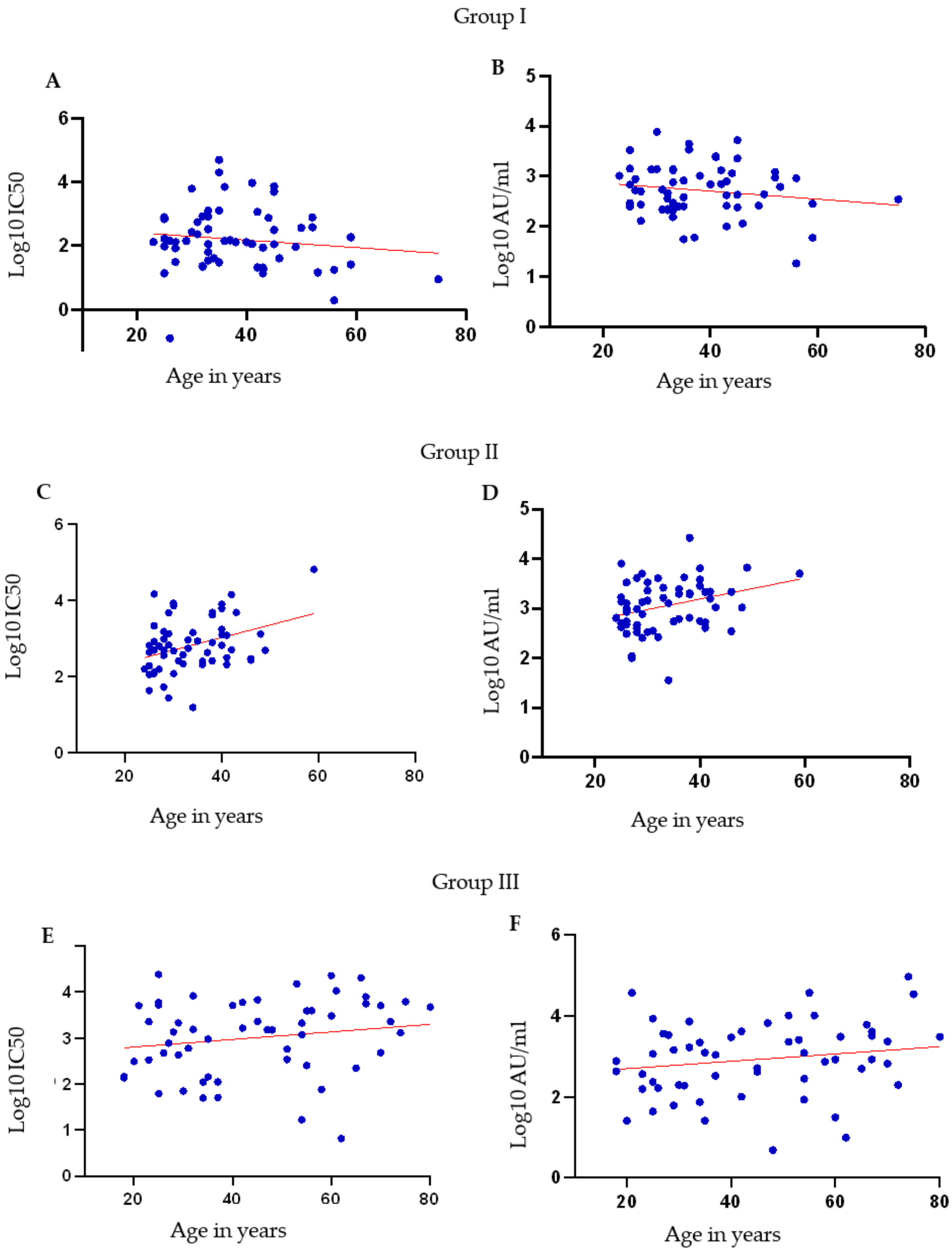

Figure S9. Correlation of antibody titers with ages in years of study participants. A, C & E indicate the correlation of log10 IC50 values obtained following pseudotyped lentivirus antibody neutralization assay with age, while B, C, & D show the correlation between log10 AU/ml values using the SARS-CoV-2 IgG II Quant immunoassay (B, D, F) and ages of participants respectively.
